# Free Hospital Patients and the Medical Profession

**Published:** 1905-02-25

**Authors:** 


### HOSPITAL ADMINISTRATION.

### CONSTRUCTION AND ECONOMICS.

### FREE HOSPITAL PATIENTS AND THE MEDICAL PROFESSION.

KING'S COLLEGE HOSPITAL, which has won so much public confidence by the decision of the Governors to remove it from Portugal Street to a crowded district of Camberwell, is thus placed in a position to provide a perfect modern hospital, up to date in every particular, including its methods of administration and the admission of patients. On the latter point the members of the Wandsworth Division of the British Medical Association have taken action through their South London Medical Committee, which was formed about a year ago, at the request of King's College Hospital, to draw up suggestions for the regulation of the admission of patients to the new hospital at Camberwell. This committee has made the following recommendations, which were considered at a meeting of the branch held on the 23rd instant, after we went to press:—

(a) That in connection with the establishment of King's College Hospital at Camberwell, there should be no issue of subscribers' letters.

(b) That sickness and poverty should be the qualifications for hospital relief.

(c) That no charge should be made for the treatment of any patient of the hospital, or for the supply of medicines or dressings.

(d) That urgent cases should receive attention without inquiry.

(e) That applicants for relief should, on their first visit, be seen by a registered medical practitioner, and, if expedient, attended to, and that the name, age, address and occupation or position should be entered in a register.

(f) That, on a second visit, the patient should bring with him a certificate, signed by a medical practitioner, stating that he (she) is a suitable person for hospital relief.

(g) That such certificate should not necessarily be written on a form supplied by the hospital.

(h) That such certificate duly filled up should be appended to each patient's prescription sheet, or attendance card.

(i) That the attached certificate be submitted to the authorities of King's College Hospital as the ordinary form to be supplied to patients attending the hospital for the first time.

(j) That it would be satisfactory if a system could be arranged at the hospital by which obscure and interesting cases might be brought or sent with a note, by practitioners in the neighbourhood, for an opinion, where a consulation fee cannot be paid.

(k) That all cases of trivial accident or illness, deemed ineligible for the out-patient department, shall, after having been once attended to, be referred for treatment to one of the local general practitioners or the neighbouring dispensaries.

(l) That should a general practitioner report in writing the attendance of a person well able to pay, the letter, if duly signed, be treated as confidential and the matter investigated.

(m) That the hospital authorities use every means in their power to prevent unsuitable cases receiving hospital treatment, and appoint such officers as they consider desirable to make inquiries with a view to checking hospital abuse.

(n) That the regulations for the treatment of patients be displayed in conspicuous places.

The question naturally arises, Can these recommendations be carried out in their entirety, and with what result? (a), (b), (d), (e), (j), (l), (m), and (n) may be readily accepted as common sense and reasonable, and will no doubt cause little or no discussion; the remainder, however, as they stand, will require elucidation and some modification if they are to remain.

#### PAYMENTS BY IN AND OUT-PATIENTS.

The recommendation that no charge should be made for the treatment of any patient of the hospital, or for the supply of medicine or dressings, is not one which in these days represents the teachings of experience or the average opinion of impartial hospital managers who understand their business. No general practitioner could fail to be benefited, for instance, by a system which provided a paying wing to which all cases that could afford to pay something for their treatment in hospital could be admitted, and where they would have the right to be attended by their own doctors. We have often elaborated this point, and need not say more upon it to-day, except to insist that all the interests affected by the treatment of paying patients shall be adequately safeguarded by the rules regulating this branch of hospital work. Turning next to the out-patients, in view of the new system of careful investigation which the above recommendations embody, there should be no practical objection to the hospital receiving a small payment from such of the out-patients as are able and willing to make it on account of the medicine which may be supplied them. Not only does this system when properly safeguarded not interfere with the private medical attendant, but it is an advantage to him in the sense that it should tend to make people less anxious to go to the out-patient department when they can have a private practitioner for a fee well within their means. It tends, too, to check the applications of hospital habitues in the out-patient department, who, as Sir William Gull used to say, seem to delight in spending their time in moving about from one hospital to another, though their ailments may be of the slightest.

#### SOCIAL FITNESS AND MEDICAL CERTIFICATES.

Recommendations (f), (g), (h), and (i) constitute a new departure in hospital relief methods, and we are doubtful if they would work in practice. Taking the whole number of medical practitioners in the district there must be amongst them a number of very busy men who would find it most distasteful, if not impossible, to have persons calling upon them for a certificate of suitability for hospital relief for which no fee could be charged, and which must entail much inquiry in addition to correspondence. It is probable that the [majority of out-patients, at any rate, are personally unknown to any medical practitioner in their neighbourhood, and how the latter are to put themselves in a position to certify on reliable evidence that such persons are suitable for hospital relief we fail to understand. The recommendations further foreshadow that every such applicant should receive a written certificate, which must increase the expenditure of time and labour of the certifying medical practitioner, and for this reason, if the plan is to be attempted, a printed form must be available. At some of the best managed special hospitals the practice has grown up of accepting a certificate of suitability from a clergyman or minister of religion, as well as from medical practitioners, so that the labour may be distributed amongst the official residents in each district, and the plan on the whole has been found to work well.

General Hospitals and General Practitioners.

We most heartily support recommendation (*j*) because we have long felt that the local doctors should be kept constantly in touch with the hospital, not only with the object of securing that obscure and interesting cases shall be sent there in consultation by the medical practitioner, but because we hold that post graduate courses at the hospitals ought to be open to all such members of the medical profession.

Hospitals to Co-operate with the Dispensaries.

As to recommendation (*a*), we have always been strongly in favour of the free and provident dispensaries working in co-operation with the hospitals. There is no system in London to-day which affords such excellent opportunities for ascertaining with accuracy the social position of patients as that of the free dispensaries. Besides, the free and provident dispensaries arrange to attend patients at their own homes, which must make the system popular and tend continually to reduce the number of out-patients at a hospital when the people in a given district have become accustomed to and understand this system of medical relief. We have no doubt that free dispensary treatment would increase greatly in popularity, but if the provident dispensary system is to take its proper place as a relief agency in the metropolis, the members of the medical profession who undertake this class of work will have to study how to make it a success. Provident dispensaries have largely failed in London in the past, because it is not sufficiently understood by members of the profession that any man who assumes office in connection with one of these institutions must so organise his consulting-room and methods of seeing patients that all members of the provident dispensary who come to him will feel that each has the best of his time, attention, and skill, and that all are always welcome to treatment when they need it. The recognition of this fact has made the Victoria Dispensary, Northampton, the huge success it is, and this success includes the receipt of large incomes by those members of the medical staff who have taken the trouble to make arrangements for carrying on the work with efficiency and despatch. We cannot imagine, however, how King's College Hospital can possibly undertake to send hundreds of cases, after having been once attended to, for treatment to one of the local general practitioners. This recommendation requires elucidation or modification.

Free Attendance at Home.

We are glad to notice that Dr. M. G. Biggs has given notice that he will move that no scheme of hospital reform will prove effectual that has not contingent to it a satisfactory arrangement for providing medical treatment in their own homes for those of the lower and lower middle classes who do not need hospital treatment, upon terms which will be satisfactory both to the general practitioner and the patient. No doubt such a system of co-operation between the hospital and the free and provident dispensaries in the district will largely supply what Dr. Biggs suggests by his scheme. Such an arrangement has been found to work successfully in connection with the Bolingbroke Hospital at Wandsworth, and we see no reason why a scheme of this nature cannot be elaborated upon such a basis as to meet the wishes and fulfil the requirements of all the interests affected. After all the result to aim at is that whilst every person who is entitled to it shall have free medical relief at our voluntary hospitals, the authorities shall so organise their system of admission as to exclude all who cannot prove their right to claim it.

A Medical Board of Reference.

Dr. Biggs will move that the committee be not dissolved until a satisfactory arrangement has been come to with King's College Hospital, and that before dissolving the committee will form a permanent watch committee. It is no doubt desirable for the smooth working of any arrangements which may be come to as a settlement satisfactory to the medical profession and the hospital committee, that provision for future co operation between these interests shall be made. We consider the suggestion of a watch committee, however, to be unfortunate in principle and in name. We would suggest, as an alternative, that representatives of the medical profession, who may or may not have been members of the South London Committee, should be selected to act as a medical board of reference. To this board all questions might be referred from time to time by the hospital authorities and by others should they have matters which they considered it desirable to raise. Under such a plan as this it should be possible to maintain the necessary touch, and at the same time to continuously promote the most friendly relations between the hospital and the profession, and so to help the smooth working of the scheme from year to year.

